# Next-Generation Sequencing Identification and Characterization of MicroRNAs in Dwarfed Citrus Trees Infected With Citrus Dwarfing Viroid in High-Density Plantings

**DOI:** 10.3389/fmicb.2021.646273

**Published:** 2021-04-30

**Authors:** Tyler Dang, Irene Lavagi-Craddock, Sohrab Bodaghi, Georgios Vidalakis

**Affiliations:** Department of Microbiology and Plant Pathology, University of California, Riverside, Riverside, CA, United States

**Keywords:** gene regulation, miRNA, sRNA, RNAi, vdsRNA, siRNA, gene silencing, plant antiviral response

## Abstract

Citrus dwarfing viroid (CDVd) induces stunting on sweet orange trees [*Citrus sinensis* (L.) Osbeck], propagated on trifoliate orange rootstock [*Citrus trifoliata* (L.), syn. *Poncirus trifoliata* (L.) Raf.]. MicroRNAs (miRNAs) are a class of non-coding small RNAs (sRNAs) that play important roles in the regulation of tree gene expression. To identify miRNAs in dwarfed citrus trees, grown in high-density plantings, and their response to CDVd infection, sRNA next-generation sequencing was performed on CDVd-infected and non-infected controls. A total of 1,290 and 628 miRNAs were identified in stem and root tissues, respectively, and among those, 60 were conserved in each of these two tissue types. Three conserved miRNAs (csi-miR479, csi-miR171b, and csi-miR156) were significantly downregulated (adjusted *p*-value < 0.05) in the stems of CDVd-infected trees compared to the non-infected controls. The three stem downregulated miRNAs are known to be involved in various physiological and developmental processes some of which may be related to the characteristic dwarfed phenotype displayed by CDVd-infected *C. sinensis* on *C. trifoliata* rootstock field trees. Only one miRNA (csi-miR535) was significantly downregulated in CDVd-infected roots and it was predicted to target genes controlling a wide range of cellular functions. Reverse transcription quantitative polymerase chain reaction analysis performed on selected miRNA targets validated the negative correlation between the expression levels of these targets and their corresponding miRNAs in CDVd-infected trees. Our results indicate that CDVd-responsive plant miRNAs play a role in regulating important citrus growth and developmental processes that may participate in the cellular changes leading to the observed citrus dwarf phenotype.

## Introduction

Small RNAs (sRNAs) can be divided into several categories, which include small-interfering (si)RNAs, *trans*-acting (ta)-siRNAs, microRNAs (miRNAs), natural-antisense siRNAs (nat-siRNAs), and Piwi-interacting RNAs (piwi-RNAs) (Borges and Martienssen, 2015; Czech et al., 2018; Zhu et al., 2018; Treiber et al., 2019). One of the major components of endogenous plant sRNAs are miRNAs. miRNAs are encoded by plant *MIR* genes and have independent transcriptional units with their own regulatory promoters. They form double stranded stem loop structures that are processed to produce single stranded transcripts, typically 21–24 nucleotides (nt) in length (Wang et al., 2019).

**FIGURE 1:**
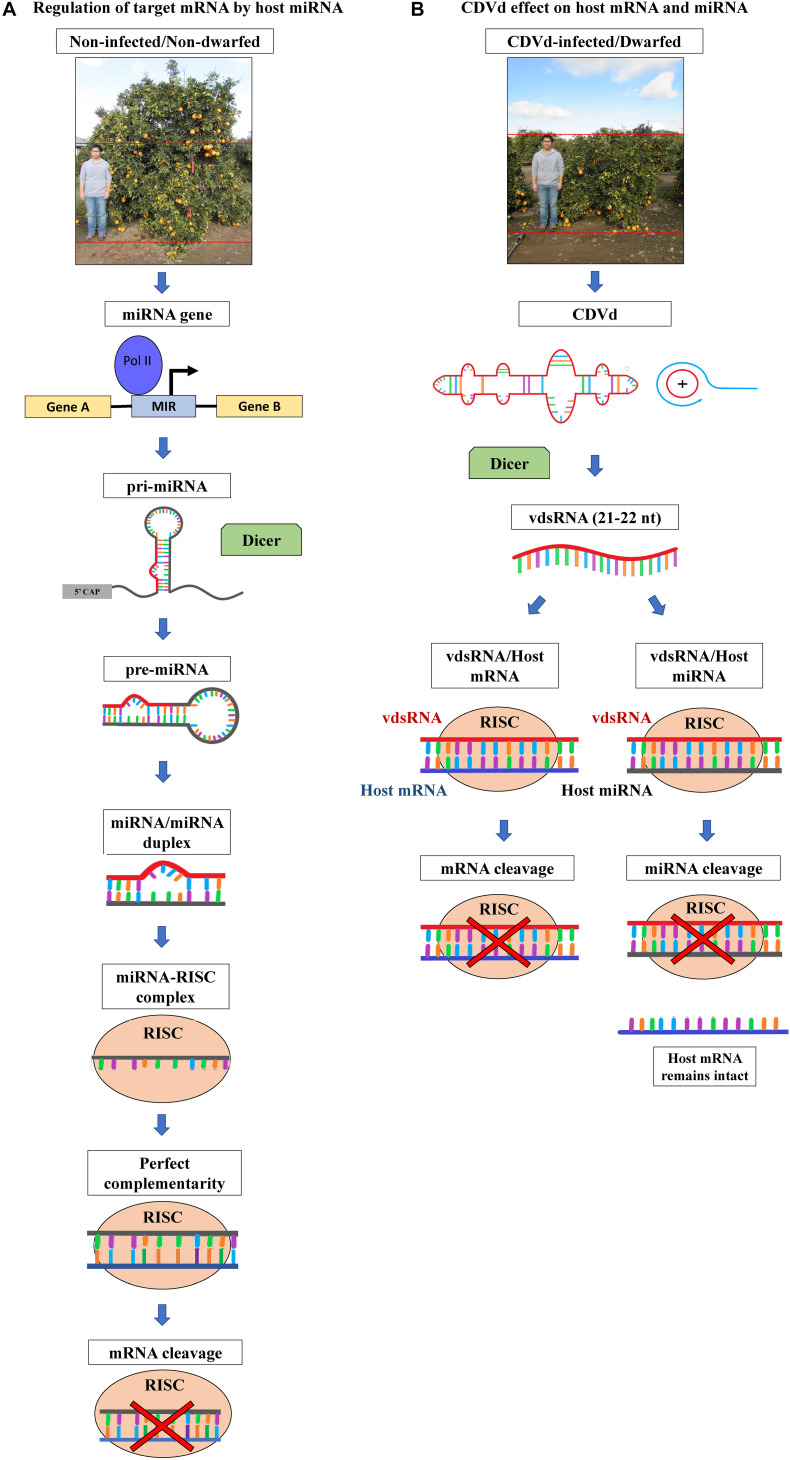
Citrus phenotypes and schematic representation of host microRNA (miRNA)-based gene expression regulatory pathway and the hypothesized effects of citrus dwarfing viroid (CDVd) on the expression of host target genes. **(A)** Non-viroid-infected, non-dwarf phenotype (∼3 m height) of navel orange [*Citrus sinensis* (L.) Osbeck] on trifoliate orange [*Citrus trifoliata* (L.) Raf.] rootstock, citrus tree and regulation of target messenger RNA (mRNA) by host miRNA. miRNA genes are transcribed by RNA polymerase II into primary miRNA (pri-miRNA) that folds into self-complementary stem-loop structures. The pri-RNA is then processed by the dicer-like (DCL) RNAse III endonucleases to form precursor miRNA (pre-miRNA), and the pre-miRNA is cleaved again to generate a miRNA/miRNA duplex. One strand of the duplex is degraded and the guide strand is incorporated into the RNA-inducing silencing effector complex (RISC). RISC, with RNAse H activity, targets RNA complementary to the miRNA guide strand. Once the complex finds the complementary plant mRNA, cleaves it, thus regulating its gene expression levels (for a review see Wang et al., 2019). **(B)** Viroid-infected, dwarf phenotype (∼2 m height) of navel orange trees on trifoliate orange rootstock and the production of viroid derived small RNAs (vdsRNAs). The highly structured CDVd RNA molecule and double stranded intermediate RNAs produced during the viroid rolling-circle replication, are processed by DCL into 21–22 nt vdsRNAs. vdsRNAs are incorporated into RISC and can be guided to form either vdsRNA/host mRNA or vdsRNA/host miRNA complexes. As a result, CDVd vdsRNAs could alter the expression levels of plant mRNA (directly or indirectly via cleavage of plant miRNAs), therefore resulting in the observed citrus tree dwarf phenotype in the field (for a review see Dadami et al., 2017).

MicroRNAs have essential functions in plant development and are involved in regulating a myriad of plant processes such as leaf, root, stem, and floral organ morphogenesis and development, biosynthesis, metabolism, homeostasis, vegetative to reproductive growth transition, senescence, signal transduction, and response to biotic and abiotic stress. Upon expression and processing, plant miRNAs are incorporated into the activated RNA-induced silencing complex (RISC) to target RNAs which are complementary to the miRNA guide strand. Once the activated miRNA–RISC complex finds the complementary plant mRNA, it silences the target via RNA degradation or translational repression (Wang et al., 2019) (Figure 1A).

Viroid derived sRNAs (vdsRNA) are products of the RNA interference (RNAi) basal plant antiviral defense response (Navarro et al., 2012; Dadami et al., 2013, 2017; Eamens et al., 2014; Adkar-Purushothama et al., 2015, 2017; Reis et al., 2015; Taliansky et al., 2021). Viroids (246–401 nt), highly structured, autonomously replicating RNA plant pathogenic agents, and trigger RNAi during their replication due to the formation of double stranded intermediate RNAs (Flores et al., 2009; Dadami et al., 2017). Similarly to plant endogenous sRNAs, vdsRNAs are 21–22 and 24 nt in length and have been detected in plants infected by several different viroids (Navarro et al., 2009; Bolduc et al., 2010; Diermann et al., 2010; Tsushima et al., 2011). vdsRNAs play an important role in viroid-mediated biological and pathogenic activities by guiding the RISC-mediated cleavage of host RNAs (Figure 1B) (Wassenegger et al., 1994; Itaya et al., 2007; Navarro et al., 2012; Dadami et al., 2013, 2017; Eamens et al., 2014; Adkar-Purushothama et al., 2015, 2017; Reis et al., 2015; Flores et al., 2017; Ramesh et al., 2020). Viroid infection might cause symptoms through the action of vdsRNAs which alter the expression levels of plant miRNAs, which in turn affects the expression levels of the plant mRNA targets of those plant miRNAs. It was reported that potato spindle tuber viroid (PSTVd) infection of tomato affects host miRNA production (Diermann et al., 2010) and host mRNA production (Wang et al., 2011; Owens et al., 2012). It was also reported, citrus bark cracking viroid infection was shown to affect plant miRNA regulation of plant transcription factors regulating leaf, cone and root growth and development of hop plants (Mishra et al., 2016).

The *Citrus* genus (family *Rutaceae*), includes several cultivars of high economic value including oranges, mandarins, grapefruits and lemons (2018–2019 US citrus crop packinghouse-door equivalent $3.35 billion) (USDA-NASS, 2019). Citrus flavors and aromas are among the most recognizable and preferred worldwide. In addition, citrus fruits are a rich source of vitamins, antioxidants, minerals, and dietary fiber essential for overall nutritional wellbeing (Van Duyn and Pivonka, 2000; Yao et al., 2004). Citrus trees are produced by grafting a desired scion variety onto a suitable rootstock species that then are planted in commercial citrus orchards. Tree spacing in citrus orchards has varied depending upon the cultivated species and a variety of factors such as soil type, climatic conditions and available farming equipment (Platt, 1973; Tucker and Wheaton, 1978). The historical global trend of citrus orchard spacing has been toward higher tree densities to maintain yield on the reduced available agricultural land and to increase economic returns.

Citrus dwarfing viroid (CDVd) infection of navel orange trees [*Citrus sinensis* (L.) Osb.] propagated on trifoliate orange [*Citrus trifoliata* (L.), syn. *Poncirus trifoliata* (L.) Raf.] rootstock has been previously reported to reduce canopy volume by approximately 50% (Figure 1B) (Vidalakis et al., 2011) and we recently demonstrated that the observed reduction in tree size results from *a* > 20% reduction in the apical growth of individual shoots within the tree canopy (Lavagi-Craddock et al., 2020). Understanding the molecular mechanism of the CDVd-induced citrus tree size reduction, will be most valuable as it could provide information on how to systematically produce dwarf trees for high density plantings.

To date, very few studies exist of miRNAs in citrus and even fewer in navel orange trees (Lu et al., 2015; Ma et al., 2016; Liang et al., 2017; Xie et al., 2017; Huang et al., 2019) and to our knowledge, there are no published studies on citrus miRNAs in response to viroid infection of citrus field trees. To explore the effect of CDVd-infection on citrus miRNAs and gain insight into the symptom development mechanism leading to the dwarfed phenotype observed in field plantings, we analyzed the effect of CDVd infection using next-generation sequencing (NGS) approach. The increasing number of miRNAs deposited in the miRBase database (Kozomara and Griffiths-Jones, 2014; Kozomara et al., 2019) from a wide range of species (<200), including *C. sinensis*, enables the discovery of novel miRNAs and their responses to pathogen infection, which may account for the observed species specific reactions and symptom development. Many plant miRNAs are conserved (Axtell and Bartel, 2005) but some are species specific (Moxon et al., 2008) and expressed at lower levels, thus making NGS the ideal approach to discover them and study their expression profiles (Jagadeeswaran et al., 2010; Motameny et al., 2010). Indeed, miRNAs from different plant species such as maize (Zhang et al., 2009), potato (Zhang et al., 2013), peanut (Zhang et al., 2017), barley (Ferdous et al., 2017), soybean (Zhang et al., 2008), and hop (Mishra et al., 2016), have been identified using NGS approaches.

In this study, we analyzed sRNA libraries prepared from field grown CDVd-infected navel orange and non-infected control trees to characterize miRNAs in the *C. sinensis* (stems) and *C. trifoliata* (roots) genomes and their expression profile in response to CDVd infection. This work provides valuable information at the molecular level and establishes the foundational framework that is necessary to dissect the subcellular mechanisms responsible for the observed citrus dwarf phenotype in the field.

## Materials and Methods

### Plant Material and RNA Isolation

Plant material (stems and roots) was collected in January 2016 from six 18-years-old “Parent Washington” navel [*C. sinensis* (L.) Osbeck] on “Rich 16-6” trifoliate orange [*C. trifoliata* (L.), syn. *Poncirus trifoliata* (L.) Raf.] rootstock infected (*n* = 3) and non-infected (*n* = 3) with CDVd, respectively. Trees were planted in an East–West running orchard located at the University of California (UC), Agriculture and Natural Resources, Lindcove Research and Extension Center (Exeter, CA, United States). CDVd-infected trees were planted at high density (3 × 6.7 m), whereas non-infected control trees were spaced at standard density (6.1 × 6.7 m).

Stem and root samples were processed in the field and immediately frozen in liquid nitrogen. For each tree, eight stem samples from around the canopy were collected. Leaves and petioles were removed, the stems were roughly chopped into approximately 0.5–1 cm pieces, placed into 50 ml conical tubes, and flash frozen. Root samples were collected from around the tree, at approximately 1 m away from the trunk and 20 cm deep, near the irrigation emitters, using a corer. The roots from eight core soil samples were washed thoroughly with water, gently blotted dry with paper towels, chopped into 0.5–1 cm pieces, placed into 50 ml conical tubes, and flash frozen. In between each sample collection and processing, cutting tools, and working surfaces were sanitized with 10% bleach solution (0.5–1% sodium hypochlorite) and rinsed with water and new sterile disposable plasticware and razor blades were used. Samples were transported into the Citrus Clonal Protection Program (CCPP), Citrus Diagnostic Therapy and Research Laboratory at the UC Riverside (Riverside, CA, United States) on dry ice and stored at −80°C until analysis.

Total RNA was isolated using the Invitrogen^TM^ TRIzol^TM^ (Thermo Fisher Scientific, Waltham, MA, United States) reagent. For each sample, 300 mg of frozen tissue were ground in liquid nitrogen with mortar and pestle. The ground material was transferred to a 5 ml Eppendorf tube and 3 ml of TRIzol^TM^ reagent was added immediately. RNA extraction was performed according to the manufacturer’s instructions. The eluted RNA was aliquoted into four 1.5 ml microcentrifuge tubes to prevent freezing-thawing cycles during downstream analysis. The RNA concentration and quality was assessed with a spectrophotometer and the Agilent 2100 Bioanalyzer (Agilent, Santa Clara, CA, United States) using the Plant RNA Nano assay (RIN values were between 7.9 and 8.6).

The presence or absence of CDVd in each sample was confirmed by reverse transcription quantitative polymerase chain reaction (RT-qPCR) using a CCPP developed and validated assay [F: 5′-AACTTACCTGTCGTCGTC-3′; R: 5′-CGTGTTTTACCCTGGAGG-3′; Probe (FAM): 5′-CTCCGCTAGTCGGAAAGACTCCGC-3′]. The assay was performed using the iTaq Universal Probes One-Step Kit (Bio-Rad, Hercules, CA, United States) in 20 μL reactions with 10 μL of iTaq universal probe reaction mix, 0.5 μL of reverse transcriptase, 0.6 μL of forward primer (300 nM final concentration), 1.2 μL reverse primer (600 nM final concentration), 0.4 μL of probe (200 nM final concentration), 1 μL of RNA template, and 6.3 μL of water. The RT-qPCR was performed in the Bio-Rad CFX-96 and the reaction conditions were as follows: 30 min at 50°C, 5 min at 95°C, followed by 45 cycles of 10 s 95°C, 30 s at 59°C.

### Next-Generation Sequencing, sRNA Library Preparation and Sequencing Analysis to Identify Conserved and Novel miRNAs and Their Predicted Targets

The sRNA libraries were prepared using the Illumina TruSeq Small RNA Kit (San Diego, CA, United States) following the manufacturer’s recommended protocol. The libraries were sequenced using an Illumina HiSeq^TM^ 2500 instrument with single-end 50 bp reads (SeqMatic, Fremont, CA, United States). Raw reads were trimmed to remove low quality bases and adapters using cutadapt v. 1.15 (Martin, 2011) to generate clean sRNAs reads ranging from 18 to 28 nt in length.

The clean reads were then filtered for rRNA, tRNA, snRNA, snoRNA, repeat sequences, and other ncRNAs, using Rfam v.13.0 (Kalvari et al., 2018) with default parameters. The remaining reads were mapped to known miRNAs from the miRBase database (release 21, June 2014) to identify conserved miRNAs (Kozomara and Griffiths-Jones, 2011, 2014). The reads were further analyzed to predict potential novel miRNAs using miR-PREFer v. 0.24 using default parameters (Lei and Sun, 2014).

The conserved and novel miRNA sequences were analyzed against *C. sinensis* mRNA transcripts and *C. trifoliata* coding sequences (CDS) using psRNATarget v. 2.0 (Dai et al., 2018) to predict potential miRNA-mRNA interactions. DESeq2 v. 1.18 (Love et al., 2014) was used for the differential expression analyses of the miRNAs. The annotated mRNA targets were identified using the Blast2Go (Götz et al., 2008) tool within the OmicsBox software suite v. 1.4.11 (Cambridge, MA, United States). Figures were created using GraphPad Prism v. 9.0 (San Diego, CA, United States).

### Expression Analysis of Citrus miRNAs and miRNA Target Genes Using RT-qPCR

To validate the expression levels of conserved and novel miRNAs, custom stem-loop RT-qPCR assays (catalog number: 4398987) were designed by Thermo Fisher Scientific based on the sequences provided in Supplementary Table 1. For the relative expression quantification, U6 spliceosomal RNA was used as an internal control gene to normalize the efficiency between the target and internal control using the comparative Cq method (Schmittgen and Livak, 2008; Kou et al., 2012). The assay was carried out based on the manufacturer’s recommended protocol and all samples were standardized to the same concentration to ensure equal representation. The reverse transcription reactions were performed in a total volume of 15 μL with the TaqMan^TM^ MicroRNA Reverse Transcription Kit (Thermo Fisher) which contained 0.15 μL of 100 mM dNTP, 1 μL of MultiScribe Reverse transcriptase, 1.5 μL of 10x RT Buffer, 0.19 μL of RNase Inhibitor, 4.16 μL of nuclease-free water, 5 μL of total RNA, and 3 μL of 5x RT primer. The reverse transcription reactions were performed with the ProFlex PCR System (Thermo Fisher) as follows: 16°C for 30 min, 42°C for 30 min, 85°C for 5 min, and 4°C hold. The endpoint qPCR was performed in triplicates, according to the MIQE guidelines (Bustin et al., 2009), on a QuantStudio 12K Flex Real-Time PCR System (Thermo Fisher) with the TaqMan^TM^ Fast Advanced Master Mix (Thermo Fisher) in a total of 20 μL reactions which included: 10 μL of master mix, 7.67 μL of nuclease-free water, 1 μL of TaqMan Small RNA Assay, 1.33 μL of the cDNA template. The endpoint PCR conditions were as follows: 50°C for 2 min, 95°C for 20 s, followed by 40 cycles of 95°C for 1 s, and 60°C for 20 s.

To verify the relative expression levels of the miRNA target genes, primers for the predicted target genes of miRNAs, were designed for RT-qPCR (Supplementary Table 2). Actin2 was used as an internal control gene to determine the relative abundance of the target mRNA expression levels by the comparative Cq method (Schmittgen and Livak, 2008; Mafra et al., 2012). Reverse transcription was performed using the Invitrogen^TM^ SuperScript^TM^ II Reverse Transcriptase (RT) (Carlsbad, CA, United States). The reaction was performed using the manufacturer’s recommended protocol as follows: 1μL of olig (dT) (500 μg/mL), 1 μL of dNTP (10 mM), 2 μL of total RNA and 8 μL of nuclease-free water. The mixture was incubated for 5 min at 65°C and subsequently chilled on ice. The reaction was prepared with 4 μL of 5x First-Strand Buffer, 2 μL of 0.1M DTT, and 1 μL of RNaseOUT (40 units/μL) and then incubated for 2 min at 42°C. Finally, 1 μL of SuperScript^TM^ II RT (200 units) was added and the reaction was incubated at 42°C for 50 min followed by 70°C for 15 min. Downstream qPCR was also performed in triplicates, according to the MIQE guidelines, using the iTaq Universal SYBR Supermix (Bio-Rad): 10 μL of iTaq Universal SYBR Supermix, 1 μL of cDNA, 0.6 μL of each forward and reverse primers and 7.8 μL of nuclease-free water. The qPCR was performed on the Bio-Rad CFX-96 with the following conditions: 95°C for 1 min, followed by 40 cycles of 95°C for 10 s, and 60°C for 15 s.

## Results

### Next-Generation Sequencing and Characterization of Potential Citrus miRNAs

To characterize citrus miRNAs and their expression profile in response to CDVd infection, we prepared and analyzed two sRNA libraries from stems and root samples of CDVd-infected and non-infected controls of navel orange citrus trees on trifoliate orange rootstock (Table 1).

**TABLE 1:** Statistical summary of small RNA (sRNA) sequences from non-infected and citrus dwarfing viroid (CDVd)-infected libraries from stem (*Citrus sinensis*) and root (*Citrus trifoliata*) tissues.

	Non-infected stem		CDVd-infected stem	
	Reads	Unique sequences	Reads	Unique sequences
Raw reads	16,008,944	N/A	13,764,218	N/A
Clean reads (18–28 nt sRNA)	6,742,931 (100%)	1,453,586 (100%)	5,733,421 (100%)	1,214,014 (100%)
miRNA	545,243 (8.1%)	683 (0.05%)	412,526 (7.2%)	607 (0.05%)
rRNA, tRNA, snRNA, and snoRNA	3,220,307 (47.8%)	115,180 (7.9%)	2,880,237 (50.2%)	109,817 (9.05%)
Unannotated	2,977,381 (44.2%)	1,336,138 (91.9%)	2,440,659 (42.6%)	1,102,588 (90.8%)

	**Non-infected root**		**CDVd-infected root**	
	**Reads**	**Unique sequences**	**Reads**	**Unique sequences**

Raw reads	6,524,898	NA	5,864,614	NA
Clean reads (18–28 nt sRNA)	2,030,419 (100%)	515,978 (100%)	1,873,309 (100%)	468,357 (100%)
miRNA	125,224 (6.1%)	313 (0.00061%)	108,262 (5.8%)	315 (0.0007%)
rRNA, tRNA, snRNA, and snoRNA	1,027,218 (50.5%)	87,435 (16.9%)	984,550 (52.5%)	81,988 (17.5%)
Unannotated	877,976 (43.2%)	427,990 (82.9%)	780,496 (41.6%)	385,868 (82.4%)

From the non-infected trees, 8.1% of the stem and 6.1% of the root were classified as miRNAs. Similarly, for the CDVd-infected stems and roots, 7.2 and 5.8% of the reads, respectively, were classified as miRNAs. The unique unannotated sequences in both the non-infected and CDVd-infected stems represented at least 90% of the total reads while for the roots they represented over 82% (Table 1). The total unique miRNA reads for both non-infected and CDVd-infected stems represented 0.05% of the reads, while both non-infected and CDVd-infected roots represented 0.0006% of the reads (Table 1).

The most common size among the total mapped miRNAs sequences ranged between 20 and 24 nt in length, with 21-nt being the predominant miRNA class across different treatments and tissue types. This is consistent with plant antiviral RNAi responses and DCL-mediated processing of dsRNA producing 21 nt siRNAs.

### Identification of Conserved miRNAs and Their Expression Profiles

The miRNA sequencing from non-infected control and CDVd-infected stems and roots identified 60 unique conserved miRNAs that ranged from 20 to 24 nt (Supplementary Tables 3, 4). Based on differential expression analysis, four conserved miRNAs (three in the stems and one in the roots) were found to be significantly altered in response to CDVd-infection (*P*-value and adjusted *P*-value < 0.05) (Table 2). Our results indicated that different members of the three miRNA families of interest had different expression levels between the non-infected and the CDVd-infected trees. The conserved miRNA families in the stems included csi-miR156, csi-miR171b, and csi-miR479, while csi-miR535 was the only conserved miRNA found in the roots. The conserved stem miRNAs were moderately more abundant compared to the conserved root miRNAs (Table 2). All four conserved miRNAs had higher expression levels in the non-infected control than the CDVd-infected trees (Table 2).

**TABLE 2:** Subsets of the conserved microRNAs (miRNAs) and their recovery profile in response to citrus dwarfing viroid (CDVd)-infection in stem (*Citrus sinensis*) and root (*C. trifoliata*) tissues.

Family	miRNA name	Sequence (5′–3′)	Length (nt)	Tissue type	Normalized value		Log 2 fold change	*P*-value	Adjusted *P*-value	Significance label
					Non-infected	CDVd-infected				
MIR156	csi-miR156	UGACAGAAGAGAGUGAGCAC	20	Stem	944.28	532.04	−0.828	0.001084	0.0412	**
MIR171	csi-miR171b	CGAGCCGAAUCAAUAUCACUC	21	Stem	433.35	264.95	−0.711	0.000740	0.0338	**
MIR171	csi-miR479	UGUGAUAUUGGUUCGGCUCAUC	22	Stem	433.35	264.95	−0.711	0.000740	0.0338	**
MIR166	csi-miR166a	UCGGACCAGGCUUCAUUCCCCC	22	Stem	225,444.12	190,251.69	−0.24	0.29	0.614	
MIR166	csi-miR166b	UCGGACCAGGCUUCAUUCCCGU	22	Stem	190,840.39	211,388.61	0.15	0.25	0.610	
MIR166	csi-miR166c	UCGGACCAGGCUUCAUUCCC	20	Stem	248,503.49	210,279.64	−0.24	0.27	0.613	
MIR166	csi-miR166d	UCGGACCAGGCUUCAUUCCCU	21	Stem	10,291.47	9,164.90	−0.17	0.35	0.645	
MIR166	csi-miR166e	UCGGACCAGGCUUCAUUCCCC	21	Stem	225,437.68	190,245.62	−0.24	0.29	0.614	
MIR396	csi-miR396a	UUCCACAGCUUUCUUGAACUG	21	Stem	4,439.016609	6,389.073731	0.53	0.01	0.10	
MIR396	csi-miR396b	UUCCACAGCUUUCUUGAACUG	21	Stem	4,625.81381	6,577.736377	0.51	0.01	0.10	
MIR396	csi-miR396c	UUCAAGAAAUCUGUGGGAAG	20	Stem	3,340.320865	2,152.155423	−0.63	0.02	0.16	
MIR399	csi-miR399a	UGCCAAAGGAGAUUUGCCCGG	21	Stem	0.82	2.04	1.47	0.22	NA	
MIR399	csi-miR399b	UGCCAAAGGAGAGUUGCCCUA	21	Stem	24.93	41.24	0.74	0.08	0.379	
MIR399	csi-miR399c	UGCCAAAGGAGAAUUGCCCUG	21	Stem	2.28	4.29	0.99	0.25	NA	
MIR399	csi-miR399d	UGCCAAAGGAGAGUUGCCCUG	21	Stem	90.63	113.30	0.33	0.32	0.644	
MIR399	csi-miR399e	UGCCAAAGGAGAAUUGCCCUG	21	Stem	2.28	3.93	0.87	0.32	NA	
MIR477	csi-miR477a	ACCUCCCUCGAAGGCUUCCAA	21	Stem	63.15	53.52	−0.23	0.57	0.750	
MIR477	csi-miR477b	CUCUCCCUCAAGGGCUUCUCU	21	Stem	1,367.46	1,020.24	−0.42	0.05	0.289	
MIR477	csi-miR477c	UCCCUCGAAGGCUUCCAAUAUA	22	Stem	63.15	53.52	−0.23	0.57	0.750	
MIR482	csi-miR482a	UCUUCCCUAUGCCUCCCAUUCC	22	Stem	1,029.05	915.31	−0.17	0.35	0.645	
MIR482	csi-miR482b	UCUUGCCCACCCCUCCCAUUCC	22	Stem	632.93	519.99	−0.28	0.06	0.316	
MIR482	csi-miR482c	UUCCCUAGUCCCCCUAUUCCUA	22	Stem	207.25	288.14	0.48	0.06	0.316	
MIR535	csi-miR535	UGACAAUGAGAGAGAGCACAC	21	Root	63.97	24.38	−1.42	0.00	0.01	**
MIR159	csi-miR159	UUUGGAUUGAAGGGAGCUCUA	21	Root	1,334.66	1,369.76	0.04	0.88	1.00	
MIR479	csi-miR479	UGUGAUAUUGGUUCGGCUCAUC	22	Root	57.62	30.32	−0.96	0.01	0.48	
MIR319	csi-miR319	UUUGGACUGAAGGGAGCUCCU	21	Root	59.43	74.55	0.30	0.40	1.00	
MIR166	csi-miR166a	UCGGACCAGGCUUCAUUCCCCC	22	Root	40,255.18	37,986.77	−0.08	0.76	0.997	
MIR166	csi-miR166b	UCGGACCAGGCUUCAUUCCCGU	22	Root	56,293.03	48,592.72	−0.21	0.37	0.997	
MIR166	csi-miR166c	UCGGACCAGGCUUCAUUCCC	20	Root	41,752.00	39,585.50	−0.08	0.78	0.997	
MIR166	csi-miR166d	UCGGACCAGGCUUCAUUCCCU	21	Root	1,451.02	1,455.03	0.01	0.98	0.997	
MIR166	csi-miR166e	UCGGACCAGGCUUCAUUCCCC	21	Root	40,249.81	37,980.07	−0.08	0.76	0.997	
MIR167	csi-miR167a	UGAAGCUGCCAGCAUGAUCUG	21	Root	196.25	146.19	−0.41	0.19	1.00	
MIR167	csi-miR167b	UGAAGCUGCCAGCAUGAUCUU	21	Root	247.06	243.45	−0.02	0.96	1.00	
MIR167	csi-miR167c	UGAAGCUGCCAGCAUGAUCUG	21	Root	35.56	32.61	−0.07	0.82	1.00	
MIR171	csi-miR171a	UUGAGCCGCGCCAAUAUCAC	20	Root	0.27	0.28	0.29	0.90	1.00	
MIR171	csi-miR171b	CGAGCCGAAUCAAUAUCACUC	21	Root	57.62	30.32	−0.96	0.01	0.48	
MIR172	csi-miR172a	AGAAUCUUGAUGAUGCUGCA	20	Root	72.88	57.79	−0.32	0.40	1.00	
MIR172	csi-miR172b	AGAAUCUUGAUGAUGCGGCAA	21	Root	0.34	0.38	0.30	0.89	1.00	
MIR172	csi-miR172c	UGGAAUCUUGAUGAUGCUGCAG	22	Root	50.84	45.38	−0.15	0.73	1.00	
MIR399	csi-miR399a	UGCCAAAGGAGAUUUGCCCGG	21	Root	0.14	0.38	−0.01	1.00	0.997	
MIR399	csi-miR399b	UGCCAAAGGAGAGUUGCCCUA	21	Root	20.99	28.18	0.37	0.46	0.997	
MIR399	csi-miR399c	UGCCAAAGGAGAAUUGCCCUG	21	Root	0.20	1.60	1.93	0.17	0.997	
MIR399	csi-miR399d	UGCCAAAGGAGAGUUGCCCUG	21	Root	37.82	42.06	0.14	0.87	0.997	
MIR399	csi-miR399e	UGCCAAAGGAGAAUUGCCCUG	21	Root	0.20	1.60	1.93	0.17	0.997	
MIR477	csi-miR477a	ACCUCCCUCGAAGGCUUCCAA	21	Root	42.54	43.27	0.01	0.99	0.997	
MIR477	csi-miR477b	CUCUCCCUCAAGGGCUUCUCU	21	Root	38.37	27.60	−0.52	0.14	0.997	
MIR477	csi-miR477c	UCCCUCGAAGGCUUCCAAUAUA	22	Root	42.54	43.27	0.01	0.99	0.997	
MIR482	csi-miR482a	UCUUCCCUAUGCCUCCCAUUCC	22	Root	71.80	65.94	−0.10	0.74	0.997	
MIR482	csi-miR482b	UCUUGCCCACCCCUCCCAUUCC	22	Root	89.33	89.17	−0.03	0.92	0.997	
MIR482	csi-miR482c	UUCCCUAGUCCCCCUAUUCCUA	22	Root	27.56	29.59	0.14	0.70	0.997	
MIR530	csi-miR530a	UGCAUUUGCACCUGCACCUUG	21	Root	0.810	0.555	−0.893	0.570	0.997	
MIR530	csi-miR530b	UGCAUUUGCACCUGCAUCUUG	21	Root	0.677	1.047	0.508	0.703	0.997	

miRNAs with statistically significant values are noted with **. The complete miRNA dataset can be found in Supplementary Tables 3–6.

Five miRNA families present in both stem and root tissues were identified: miR166, miR171b, miR399, miR477, and miR482. In stems, the highest represented miRNA families were miR166, and miR399, with five members each, followed by miR171b with four members, miR396, miR477, and miR482 with three members and the remaining 40 miRNAs were represented by a single member (Supplementary Table 3). In the roots, two miRNA families (miR166 and miR399) were represented by five members, five miRNA families (miR167, miR172, miR396, miR477, and miR482) were represented by three members, two miRNA families (miR530 and miR171b) contained two members, and the remaining 40 root miRNA families were represented by a single member (Supplementary Table 4).

**FIGURE 2:**
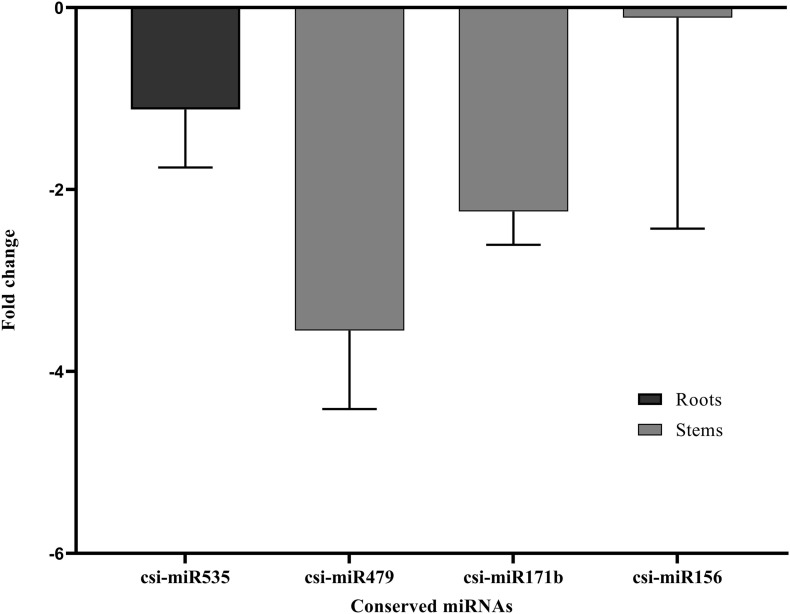
Differential expression analysis of four identified conserved miRNAs in response to CDVd infection. The relative abundance of each analyzed miRNA in CDVd-infected and non-infected trees was determined using the comparative Cq method by normalization to the U6 spliceosomal RNA. Conserved miRNAs with a significant change (*P*-value and adjusted *P*-value < 0.05) were considered to be differentially expressed. The bar graph shows the log_2_ fold change of expression levels of the miRNAs in CDVd-infected samples relative to non-infected samples in stem and root tissues.

Stem-loop RT-qPCR analysis was performed on the four conserved miRNAs in root and stem tissues (csi-miR479, csi-miR156, csi-miR171b, and csi-miR535) from non-infected and CDVd-infected trees to determine their relative abundance. The expression levels of the four conserved miRNAs were significantly altered as a result of CDVd infection. In the stems, csi-miR479’s expression decreased 3.55 fold and csi-miR171b had a fold decrease of 2.24, while csi-miR156 had the smallest negative fold change (0.11) (Figure 2). In the roots, csi-miR535′s expression decreased 1.12 fold (Figure 2). The results obtained from the RT-qPCR analysis (Figure 2) were consistent with the NGS read frequencies (Table 2) indicating a strong correlation between the RT-qPCR analysis and read frequencies obtained through small sRNA sequencing.

### Identification of Novel miRNAs and Their Expression Profiles

The lengths of the predicted novel miRNAs from stems and roots ranged between 19 and 24 nt. A total of 646 stem and 108 root novel miRNAs were identified (Supplementary Tables 5, 6). No novel root miRNAs had significant differential expression levels. On the other hand, three novel stem miRNAs (csi-miRNA-75, csi-miRNA-114, and csi-miRNA-435) had significantly different expression levels in response to CDVd infection (*P*-value and adjusted *P*-value < 0.05). All three novel stem miRNAs had higher expression levels in the non-infected trees than in the CDVd-infected trees (Table 3).

**TABLE 3:** Subsets of the novel microRNAs (miRNAs) and their recovery profile in response to citrus dwarfing viroid (CDVd)-infection in stem (*Citrus sinensis*) and root (*C. trifoliata*) tissues.

miRNA name	Sequence (5′–3′)	Length (nt)	Tissue type	Normalized value		Log 2 fold change	*P-*value	Adjusted *P*-value	Significance label
				Non-infected	CDVd-infected				
csi-miRNA-75	GUGACAGAAGAGAGUGAGCAC	21	Stem	82.35	35.66	−1.219	0.000074	0.0084	**
csi-miRNA-114	UUGGGCUCUCUUCCUCUCAUG	21	Stem	30.71	6.73	−2.247	0.000023	0.0052	**
csi-miRNA-435	GUCCCUCUCACAGCUACAGUACCC	24	Stem	45.62	23.46	−0.984	0.000380	0.0289	**
csi-miRNA-02	AAAAGGAGGACUAAGUUAAAAGCA	24	Stem	77.68	59.82	−0.39	0.08	0.38	
csi-miRNA-23	AUAUUGGAGUGUUUGACCAGU	21	Stem	60.26	46.28	−0.38	0.14	0.53	
csi-miRNA-87	ACAAGAGUUUGUGACUGUAUCAUU	24	Stem	8.55	10.60	0.20	0.67	1.00	
csi-miRNA-20	GUGACAGAAGAGAGUGAGCAC	21	Root	48.73	39.25	−0.29	0.44	1.00	
csi-miRNA-57	AUUCCUCAUUGUUUGGUCAACAGC	24	Root	13.49	9.75	−0.42	0.41	1.00	
csi-miRNA-63	UUCCAAAGGGAUCGCAUUGAUC	22	Root	59.08	53.41	−0.17	0.59	1.00	
csi-miRNA-77	AGGCAGUCUCCUUGGCUAAG	20	Root	9.54	5.30	−0.90	0.08	1.00	
csi-miRNA-91	AAGCACGAGAGAAAGACGAGAGAA	24	Root	5.21	5.17	−0.05	0.94	1.00	
csi-miRNA-100	AUUUCGGUAACUAAUAGGAUAUAC	24	Root	3.48	3.24	−0.07	0.93	1.00	

miRNAs with statistically significant values are noted with **. The complete miRNA dataset can be found in Supplementary Tables 3–6.

**FIGURE 3:**
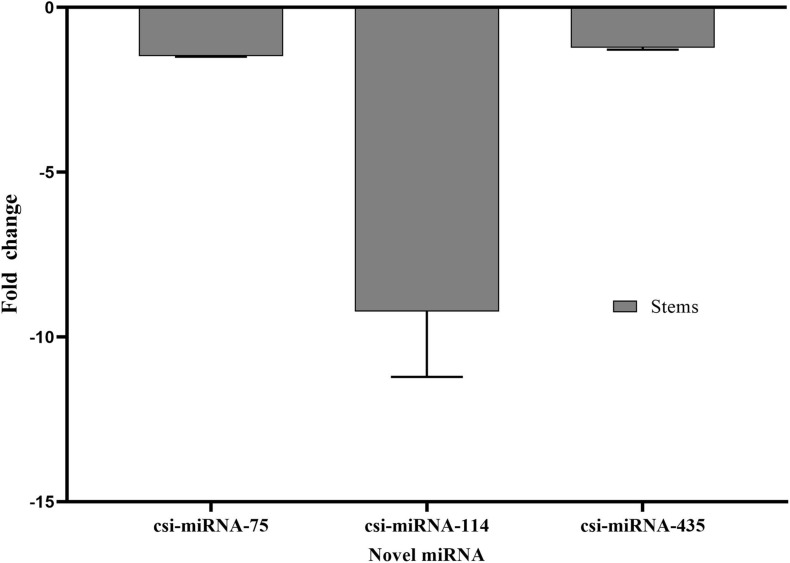
Differential expression analysis of three predicted novel miRNAs in response to CDVd infection. The relative abundance of each analyzed miRNA in CDVd-infected compared to non-infected trees was determined using the comparative Cq method by normalization to the U6 spliceosomal RNA (U6). Novel miRNAs with significant fold changes (*P*-value and adjusted *P*-value < 0.05) were considered to be differentially expressed. The bar graph shows log_2_ fold changes in expression levels of miRNAs in CDVd-infected samples relative to non-infected samples from stems.

Stem-loop RT-qPCR was performed to confirm the NGS read frequency results of the identified novel miRNAs in response to CDVd-infection. csi-miRNA-114 showed the largest expression fold change (−9.23), while csi-miRNA-75 and csi-miRNA-435 showed similar fold changes (−1.44 and −1.22, respectively) (Figure 3). These results also support the reliability of RT-qPCR (Figure 3) and NGS read frequencies (Table 3) as measures of the expression levels of miRNAs.

### Citrus miRNA-Target Prediction and Functional Analysis

To understand the function of the identified citrus miRNAs, host target genes were analyzed using the psRNATarget program by cross referencing the results against the *C. sinensis* genome for the stems (ref: GCF_000317415.1) and the *C. trifoliata* CDS for the roots (Kawahara et al., 2020). For both conserved and novel stem miRNAs, 83.1% of the miRNA targets were predicted to be regulated by cleavage and 16.9% by translational inhibition (Supplementary Tables 7, 8). Similarly, in the roots, 86.4% of the miRNA targets were predicted to be regulated by cleavage and 13.6% by translational inhibition (Supplementary Tables 9, 10).

Based on the extent of sequence complementarity between miRNAs and their targets, a total of 5,542 potential targets were predicted for the conserved and novel stem and root miRNAs (conserved: stem 63 and root 64; novel: stem 647 and root 109). Of the 5,542 potential miRNA target genes, 494 and 3,926 were targets of the conserved and novel stem miRNAs while 495 and 627 were targets of the conserved and novel root miRNAs, respectively.

The conserved stem and root miRNAs (miR479, mi171b, miR156, and miR535) analyzed in this study were associated with 10 different groups of target genes while the novel stem miRNAs (csi-miRNA-75, csi-miRNA-114, and csi-miR435) associated with three different target genes (see section “Discussion”) (Supplementary Table 11).

**FIGURE 4:**
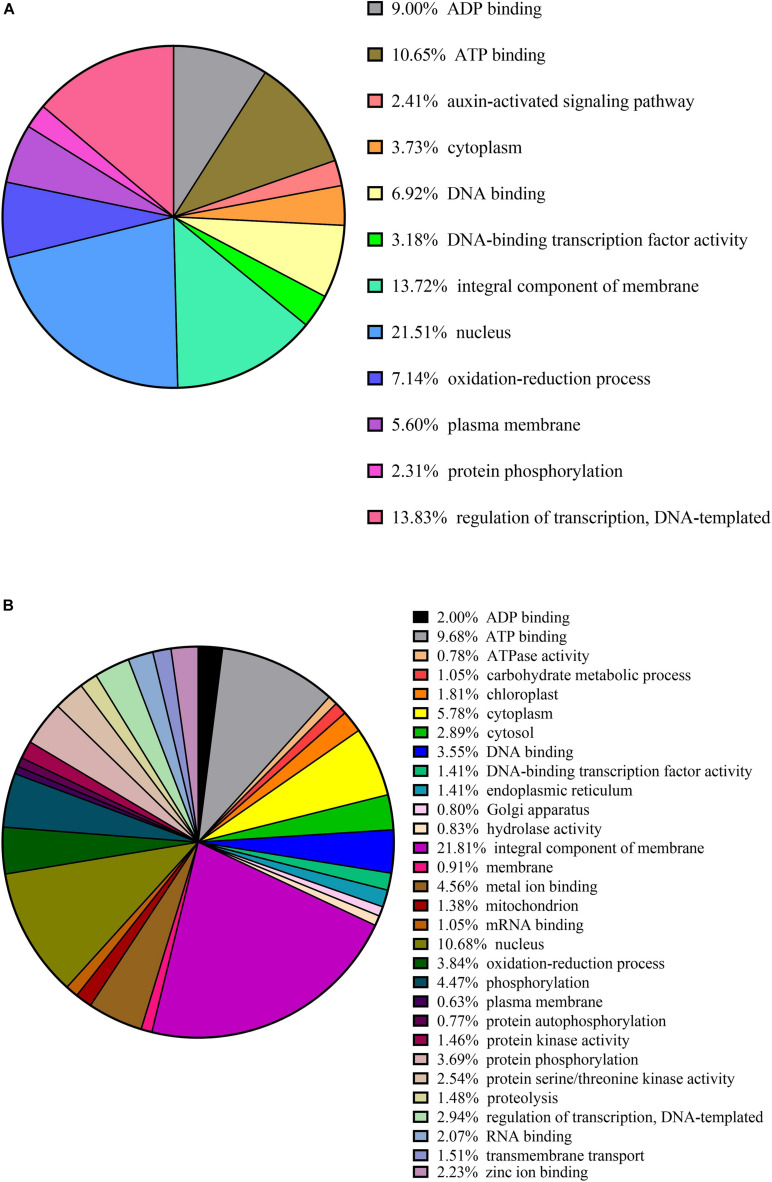
Cluster orthologous groups (COG) function calcification of predicted citrus target genes of conserved in panel **(A)** and novel in panel **(B)** miRNAs.

Clusters of orthologous groups (COG) functional classification of the targets of conserved and novel miRNAs revealed that the highest proportion of the genes were associated with (i) the nucleus (21% conserved and 11% novel); (ii) the integral component of membrane (14% conserved and 22% novel); and (iii) ATP binding (11% conserved and 10% novel) (Figures 4A,B).

Other miRNA targets shared by the conserved and novel miRNAs include (i) ADP binding (9% conserved and 2% novel); (ii) cytoplasm (4% conserved and 6% novel); (iii) DNA-binding transcription factor activity (3% conserved and 1% novel); (vi) oxidation-reduction processes (7% conserved and 4% novel); (v) plasma membrane (6% conserved and 1% novel); (vi) protein phosphorylation (2% conserved and 4% novel); and (vii) regulation of transcription (14% conserved and 3% novel) (Figures 4A,B).

**FIGURE 5:**
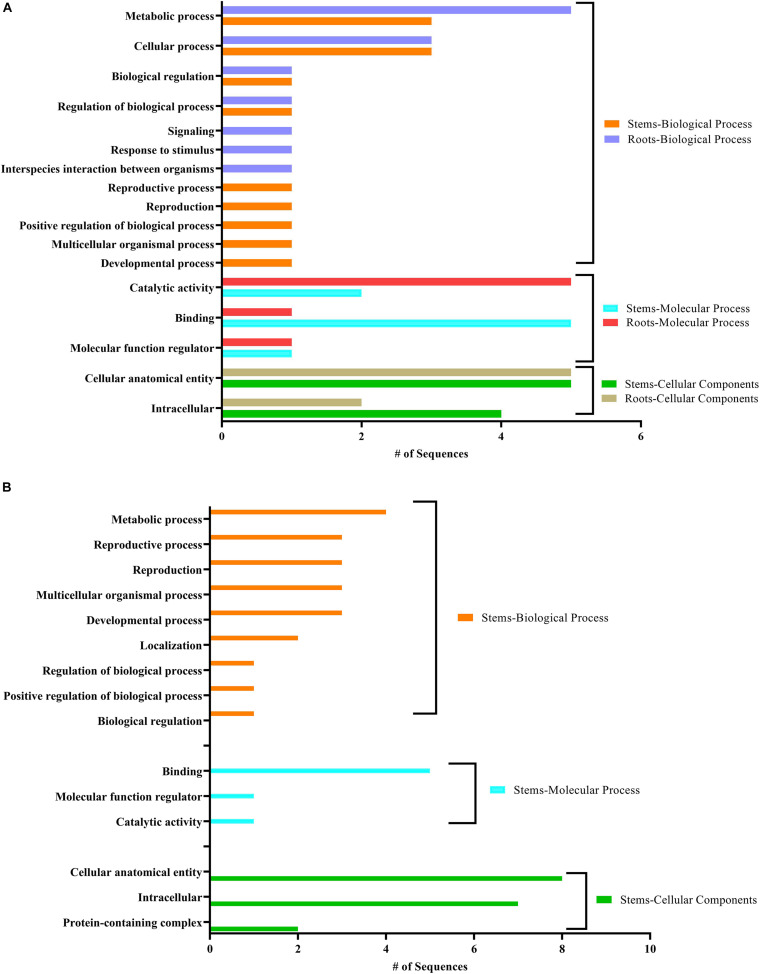
Clusters of orthologous groups functional classification of predicted target genes of conserved in panel **(A)** and novel in panel **(B)** CDVd-responsive miRNAs. The bar graph shows the number of sequences and distribution in different functional categories of the predicted miRNA targets at gene ontology (GO) level 2.

Our data were further annotated based on ontological definitions of the gene ontology (GO) terms, which categorized the predicted targets of the conserved miRNAs differentially expressed in response to CDVd infection into various biological, molecular and cellular processes (Figure 5A). Under the biological process, the predicted targets of conserved miRNA responsive to CDVd infection were subcategorized to (i) metabolic process; (ii) cellular process; (iii) biological regulation; and (iv) regulation of biological processes. The number of sequences associated with these four biological process subcategories had similar values in the stems and roots with the exception of the metabolic process that was higher in the roots (Figure 5A). For the molecular process, the majority of the predicted target genes of conserved miRNAs responsive to CDVd infection in the roots were subcategorized to catalytic activity while the targets in the stems were mostly subcategorized to binding. Targets belonging to the cellular components category were subcategorized to (i) cellular anatomical entity; and (ii) intracellular subcategories (Figure 5A) (Supplementary Table 11).

The targets of the predicted novel miRNAs displaying significant differential expression in response to CDVd infection (csi-miRNA-75, csi-miRNA-114, and csi-miRNA-435) were categorized to (i) biological process; (ii) molecular process; and (iii) cellular components. The cellular components subcategories (i) cellular anatomical entity; and (ii) intracellular contained most of the sequences (Figure 5B) (Supplementary Table 11).

### Expression Profiles and Experimental Validation of miRNA Target Transcripts

**FIGURE 6:**
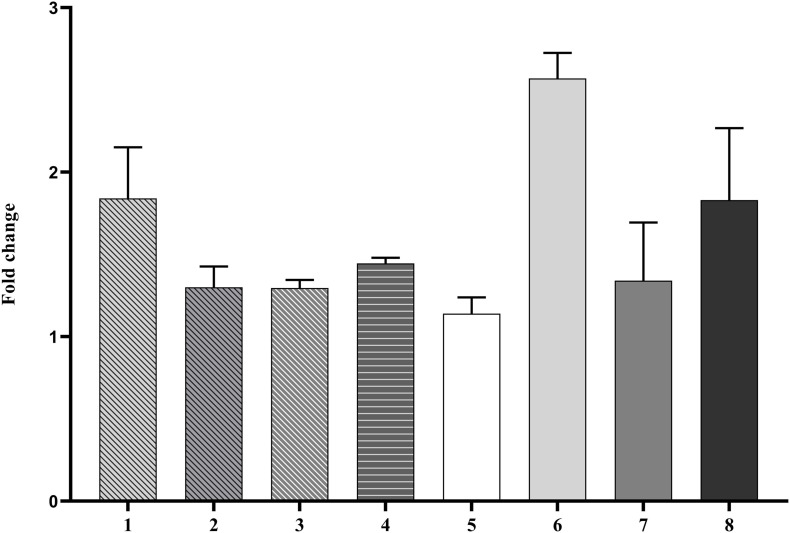
Differential expression profile of selected miRNA target genes. The relative gene expression was evaluated by the comparative Cq method using actin2 as a reference gene. The bar graph shows log_2_ fold changes of expression levels of target genes in CDVd-infected stems and roots relative to non-infected tissues. The predicted target genes used in the analysis were (1) UDP-glucose flavonoid glucosyl-transferase (orange1.1g033614m, target of csi-miR479-1-stem); (2) DEAD/DEAH box helicase (orange1.1g028826m, target of csi-miR479-2-stem); (3) DEAD/DEAH box helicase (orange1.1g026925m, target of csi-miR479-3-stem); (4) glutathione S-transferase (orange1.1g033674m, target of csi-miR171b-stem); (5) squamosa promoter binding protein-like 2 (orange1.1g011651m, target of csi-miR156-1-stem); (6) squamosa promoter binding protein-like 3 (orange1.1g032310m, target of csi-miR156-2-stem); (7) squamosa promoter-binding protein-like transcription factor family protein (orange1.1g008776m, target of csi-miR156-3-stem); and (8) RHOMBOID-like protein, P_trifoliata_00066_mRNA_51.1, target of csi-miR535-root).

The expression levels of eight predicted mRNA targets of the conserved CDVd-responsive miRNAs was determined via RT-qPCR. The results indicate that the expression of the miRNA target genes correlates negatively with the expression of their corresponding miRNA (Figure 6), thus confirming the relationship between CDVd-infection and altered expression levels of specific miRNA targets. Targets of miRNAs belonging to the same miRNA family showed variable results. For example, orange1.1g011651m (Figure 6, bar #5), orange1.1g032310m (Figure 6, bar #6), and orange1.1g008776m (Figure 6, bar #7), which are all targets of different members of the csi-miR156 family, were not uniform and showed fold-change differences.

## Discussion

Citrus production exceeded 157.9 million tons in over 9.8 million hectares worldwide for 2019^1^, while in California alone the citrus industry is valued at $3.4 billion dollars with an estimated total economic impact of $7.1 billion (Babcock, 2018). Global decrease in farmland availability, increasing land, water and labor costs, and the continued spread of the deadly Huanglongbing (HLB) disease of citrus, make it imperative to develop tools that allow for high-density citrus plantings for maximization of yields and economic returns per land surface unit. In addition, these factors have forced the citrus industry toward the implementation of novel cultivation practices that would allow for mechanized citrus production under protective structures (Gottwald, 2010; Vidalakis et al., 2011; Lambin, 2012; Verburg et al., 2013; da Graça et al., 2016).

The idea of using “graft-transmissible dwarfing agents” for tree size reduction has been continuously investigated in citrus since originally proposed by Cohen (1968) and Mendel (1968) and countries such as Australia and Israel have explored the application of such technology in commercial settings (Broadbent et al., 1992; Bar-Joseph, 1993; Hutton et al., 2000; Semancik, 2003). The observation that CDVd significantly reduced *C. sinensis* canopy volume on *C. trifoliata* rootstock (Semancik et al., 1997; Vidalakis et al., 2011); by reducing vegetative growth (Lavagi-Craddock et al., 2020) indicated that CDVd may be used as a possible tool for high-density plantings of citrus, and provided key information on the possible biological mechanism through which CDVd affects specific rootstock-scion combinations to reduce tree canopy volume (Vidalakis et al., 2011). Furthermore, understanding the detailed molecular regulatory mechanisms that lead to a reduction in tree canopy volume in response to CDVd infection would provide the necessary knowledge to produce reduced-size citrus trees without the need of a -graft-transmissible viroid agent.

Small RNAs play an essential regulatory role in cellular and plant development functions including antiviral host responses and potentially viroid pathogenesis (Borges and Martienssen, 2015; Dadami et al., 2017; Flores et al., 2017; Czech et al., 2018; Zhu et al., 2018; Treiber et al., 2019; Wang et al., 2019; Ramesh et al., 2020). Two models have been proposed to explain the involvement of RNAi in the pathogenic process induced by viroid infections and both involve vdsRNAs. In the first model, vdsRNAs might act as miRNAs, downregulating the expression of physiologically important host genes, thus inducing disease associated symptoms. vdsRNAs are expected to contain significant identity to a region of the host genome for this model to work and resistance of viroids to RNAi is a feature of the viroid genome (Wang et al., 2004). According to the second model, disease symptoms caused by the nucleus replicating pospiviroids might result from the incorporation of viroid replication intermediates into the trans-acting small interfering RNA (ta-siRNA) biogenesis pathway. The nucleolus is a ta-siRNA free zone, and mature viroid forms produced in the nucleolus are resistant to degradation. In contrast, (vd)ta-siRNA produced in the nucleus from replication intermediates can then translocate to the cytoplasm where they guide the cleavage of target host mRNA leading to observed symptoms (Gómez et al., 2009). Both models involve viroid secondary structures as a key element that can therefore be interpreted as the evolutionary compromise between the need to interact with host factors and the necessity to survive RNAi. Regardless of whether vdsRNAs are produced according to the first or second model, it is also important to point out that rather than acting directly on host mRNA, vdsRNAs may affect host mRNA targets genes via host miRNAs as previously described (Mishra et al., 2016; Dadami et al., 2017).

Plant disease resistance gene families are typically very large with thousands of members and are commonly considered the putative targets of sRNAs (Chen et al., 2010), thus making the study of sRNAs in response to viroid infection a valid approach to investigate the biological mechanisms associated with symptoms. The systematic profiling of sRNAs in CDVd-infected trees, using NGS technologies, was the next logical step to gain insight into the function and regulatory mechanisms of miRNAs through which CDVd may reduce tree canopy size. In this study, we identified conserved and novel miRNAs in citrus and their response to CDVd infection. Consistently with the distribution patterns of sRNAs in other plant species, most sRNAs from both the CDVd-infected and non-infected libraries were found in the 21 and 24 nt classes (Jia et al., 2014; Gao et al., 2015; Mishra et al., 2016; Farooq et al., 2017; Zhang et al., 2018). CDVd-infected stems produced higher frequencies of the 21-nt class than their non-infected counterparts, indicating a CDVd induction of the 21-nt class since the other sRNA classes remained at comparable levels with the non-infected libraries. The increased abundance of the 21-nt class of sRNAs in response to viroid infection observed here is in agreement with previous reports for viral (viroid and virus) infections (Minoia et al., 2014; Zavallo et al., 2015).

All identified differentially expressed conserved and novel miRNAs, in this study, displayed overall reduced expression levels in response to CDVd-infection (Figures 2, 3). Several evolutionary deeply-conserved miRNAs have been shown to retain homologous targets across plant phyla (Axtell et al., 2007) and these include miR156 (stem), miR535 (roots), and miR171b (stem), which represent three out of the four conserved miRNA with differential expression levels in response to CDVd infection identified in this study. In agreement with previous studies, miR156 was shown here to direct the cleavage of squamosa-promoter binding-like protein (SBP) box genes (orange1.1g011651m; orange1.1g032310m; and orange1.1g008776m) (Cardon et al., 1999; Rhoades et al., 2002; Wu and Poethig, 2006; Xie et al., 2006; Gandikota et al., 2007; Riese et al., 2007) (Supplementary Table 7 and Figure 6). Members of this transcription factor family are known to play important roles in flower and fruit development, plant architecture, and in the transitions from juvenile to adult stages and to flowering (Chen et al., 2010; Yu et al., 2012). Even though miR535 is also known to target squamosa promoter-binding-like protein 3 (Shi et al., 2017; Zhou et al., 2020), our study identified rhomboid-like protein 1 as the target of miR535 in the roots (P_trifoliata_00066_mRNA_51.1). In *Arabidopsis*, a rhomboid-like protein was identified, providing evidence for the existence of regulated intramembrane proteolysis (RIP), a fundamental mechanism for controlling a wide range of cellular functions, in plants (Kanaoka et al., 2005). miR171b directs the cleavage of GRAS domain transcription factor genes (Ma et al., 2014). However, in our study, we found that miR171b’s target, a probable glutathione-S-transferase, was altered in response to CDVd infection (orange1.1g033674m). Glutathione-S-transferases are ubiquitous and multifunctional enzymes encoded by large gene families that can be highly induced by biotic stress including bacterial, fungal, and viral infection (Gullner et al., 2018). We found that the less conserved miR479 cleaves the UDP-glucose flavonoid glucosyl-transferase (orange1.1g033614m) (Vogt and Jones, 2000; Offen et al., 2006) which is involved in the process of conjugating hormones, stabilizing secondary metabolites, solubility, transport, and regulating bioavailability of compounds for other metabolic process in *Arabidopsis thaliana* and DEAD/DEAH box helicases which play an important in regulatory events such as organ maturation and cellular growth and differentiation (orange1.1g028826m and orange1.1g026925m) (Macovei et al., 2012). Both target genes may be related to the observed dwarf phenotype (Supplementary Table 11).

The predicted target genes of the novel miRNAs identified in this study (csi-miRNA-75, csi-miRNA-114, and csi-miRNA-435) include proteins were associated with target genes including SBP proteins (orange1.1g030599m, orange1.1g029650m, orange1.1g021420m, orange1.1g017256m, orange1.1g016971m, orange1.1g032310m orange1.1g046416m, orange1.1g011662m, orange1.1g010865m, and orange1.1g010591m; target of csi-miRNA-75); plastid-lipid associated protein (orange1.1g030218m, orange1.1g025746m, orange1.1g030180m, and orange1.1g020639m; target of csi-miRNA-114) which are structures that contain lipids and proteins that sequester the overaccumulation of carotenoids during flower development and fruit ripening (Moriguchi et al., 1998; Leitner-Dagan et al., 2006); and vacuolar protein sorting-associated proteins (orange1.1g021304m and orange1.1g017530m; target of csi-miR435), which direct protein cargo from the Golgi apparatus to the vacuoles (Xiang et al., 2013) and have been shown to be important in plant development (Cai et al., 2014) (Supplementary Table 11). The results suggest that CDVd-infection affects a wide range of biological functions via different miRNAs. In addition, the GO distribution analysis performed in this study, identified targets of conserved and novel CDVd-responsive miRNAs involved in various processes (Figure 5). Taken together, our findings might suggest that CDVd-infection could lead to developmental reprogramming and growth alterations of citrus trees, leading to the observed symptoms of reduced vegetative growth and overall smaller tree size. Future transcriptome studies could provide additional evidence to elucidate the molecular details in support of this hypothesis.

In this study, the overall miRNA profile of roots (trifoliate orange, *C. trifoliata*) was not altered in response to CDVd infection to the same extent demonstrated by the stem (navel orange, *C. sinensis*) miRNA profile. Although CDVd-derived sRNAs were detected in the roots, the trifoliate orange rootstock does not display major symptoms in response to CDVd infection (Vidalakis et al., 2004; Vernière et al., 2006; Murcia et al., 2009). This observation is consistent with the findings that the striking dwarfed citrus tree phenotype caused by CDVd infection results from the reduced vegetative growth of the stems, supporting the hypothesis that the molecular mechanisms responsible for this reprograming must be primarily active in the stems. Future greenhouse studies could provide additional evidence to support this hypothesis.

Finally, the term “Transmissible small nuclear ribonucleic acids” (TsnRNAs) was coined to identify those viroids that do not express a disease syndrome, but rather act as modifying agents of tree performance that result in desirable agronomic traits with potential economic advantages (Semancik et al., 1997; Semancik, 2003). In our study, we did not observe induction and regulation of defense genes via the sRNA pathway in response to CDVd infection, a finding that concurs with the hypothesis that some viroid species might be considered as RNAs modifying cellular functions and plant performance.

## Conclusion

The expression profile of CDVd-responsive miRNAs indicates that these miRNAs play a role in regulating important citrus tree growth and development processes that may participate in the cellular changes leading to the observed *C. sinensis* on *C. trifoliata* rootstock dwarf phenotype.

## Data Availability Statement

The datasets presented in this study can be found in the NCBI Sequence Read Archive under accession numbers SRS8100788-SRS8100791 and PRJNA693870.

## Author Contributions

TD, IL-C, and GV conceived and designed the experiments, analyzed the data, and wrote the manuscript. TD and IL-C performed the experiments with the assistance of SB. All authors contributed to the article and approved the submitted version.

## Conflict of Interest

The authors declare that the research was conducted in the absence of any commercial or financial relationships that could be construed as a potential conflict of interest.

## Footnotes

1 http://www.fao.org/faostat/en/#data/QC

## Supplementary Material

Supplementary Table 1 Sequences submitted for stem-loop RT-qPCR of miRNAs.

Supplementary Table 2 Specific primers used for RT-qPCR relative quantification of miRNA target genes.

Supplementary Table 3 List of all conserved miRNAs found in *Citrus sinensis* stems.

Supplementary Table 4 List of all conserved miRNAs found in *Citrus trifoliata* roots.

Supplementary Table 5 List of all novel miRNAs found in stems.

Supplementary Table 6 List of all novel miRNAs found in roots.

Supplementary Table 7 List of predicted targets for conserved miRNAs in stems.

Supplementary Table 8 List of predicted targets for novel miRNAs in stems.

Supplementary Table 9 List of predicted targets for conserved miRNA in roots.

Supplementary Table 10 List of predicted targets for novel miRNAs in roots.

Supplementary Table 11 List of target genes of citrus dwarfing viroid-responsive conserved and novel miRNAs.
